# Sarcopenia in liver cirrhosis: perspectives from epigenetics and microbiota

**DOI:** 10.3389/fmed.2023.1264205

**Published:** 2023-10-10

**Authors:** Jia-qi Xu, Yu-ke Pan, Jie-xin Zhang, Shi-xue Dai, Li-shu Xu

**Affiliations:** ^1^The First School of Clinical Medicine, Southern Medical University, Guangzhou, China; ^2^Department of Joint Surgery, Orthopedic Hospital of Guangdong Province, The Third Affiliated Hospital of Southern Medical University, Guangzhou, China; ^3^The Third School of Clinical Medicine, Southern Medical University, Guangzhou, China; ^4^Department of Gastroenterology, Guangdong Provincial Geriatrics Institute, National Key Clinical Specialty, Guangdong Provincial People's Hospital (Guangdong Academy of Medical Sciences), Southern Medical University, Guangzhou, China; ^5^Department of Gastroenterology, Geriatric Center, National Regional Medical Center, Ganzhou Hospital Affiliated to Guangdong Provincial People's Hospital (Guangdong Academy of Medical Sciences), Southern Medical University, Ganzhou, China; ^6^The Second School of Clinical Medicine, Southern Medical University, Guangzhou, China

**Keywords:** sarcopenia, liver cirrhosis, epigenetics, dysbiosis, microecological interventions

## Abstract

Sarcopenia is characterized by the loss of muscle mass and function. It is well known that sarcopenia is often associated with aging, while in recent years, sarcopenia comorbid with chronic diseases such as cirrhosis has attracted widespread attention, whose underlying molecular mechanisms remain unclear. Since cirrhosis and sarcopenia are assumed to be closely interrelated in terms of pathogenesis, this review innovatively discussed the role of epigenetic modifications and microecological dysregulation in sarcopenia in the context of liver cirrhosis. Here we illustrated the relationship between sarcopenia and cirrhosis in the aspect of epigenetics, dysbiosis, and the crosstalk between gene modifications and intestinal microecology. Furthermore, the alterations in cirrhosis patients with sarcopenia, such as inflammatory response and oxidative stress, are found to present synergistic effects in the pathways of epigenetics and dysbiosis leading to sarcopenia. This review proposes that microbiome-based therapies are promising to break the vicious cycle between epigenetic modification and dysbiosis, providing strong support for the use of intestinal microecological interventions to prevent sarcopenia in cirrhotic patients.

## 1. Introduction

Sarcopenia is a progressive, pervasive disorder of skeletal muscle, which increases the incidence of events such as falls, weakness, and death, leading to a poor prognosis for patients (1). There are multiple criteria for the diagnosis and classification of sarcopenia, with EWGSOP and AWGS being the most used classification systems. The prevalence of sarcopenia varies widely depending on the classification system and cut-off point, ranging from 10 to 27% in meta-analyses (2). Age is generally considered to be the most significant factor causing sarcopenia, while risk factors brought by genetic changes, environment, lifestyle, and other diseases also play an important role (3).

Of the various risk factors, liver cirrhosis has been proven highly correlated with sarcopenia due to poor nutrition and hormone level changes. A recent meta-analysis (4), which involved 22 studies containing nearly 7,000 patients with cirrhosis, statistically concluded that the overall probability of developing sarcopenia in patients with cirrhosis was 37.5%. As is also presented in this study, the incidence of sarcopenia increased with higher Child-Pugh scores, with more than half of the Child-Pugh class C patients combined with sarcopenia. In addition, the study proposed that sarcopenia was an independent risk factor for death in patients with cirrhosis. After excluding other risk factors for death, such as hepatocellular carcinoma, the mortality rate of patients with sarcopenia can still be more than double that of patients without sarcopenia.

Previous studies have suggested that the causes of sarcopenia in cirrhosis include subjective reduction in physical exercise and nutrient intake. However, certain pathophysiologic changes in cirrhosis, such as hepatocellular necrosis and portal shunts, can lead to a high release of cytokines, hyperammonemia, and endotoxemia, further resulting in more serious anabolic resistance. According to Tandon et al. (5), such a situation can prevent the patient from stimulating a normal increase in protein synthesis and decrease in hydrolysis, even with enhanced nutrition and exercise. These clues suggest that this common complication of cirrhosis might have been overlooked, and some deeper connections between cirrhosis and sarcopenia could have been ignored.

Under a large volume of literature review, we are further concerned that specific epigenetic alterations have been observed in patients with cirrhosis, such as upregulation of myostatin resulting from hyperammonemia (6), and the activation or inactivation of hepatic stellate cells (HSCs) mediated by numerous miRNAs (7), which are highly correlated with cirrhosis. Meanwhile, it is also found that patients with liver cirrhosis have intestinal microecological dysregulation, including a less healthy gut microbiome, the loss of intestinal flora richness (8), an impaired barrier function of the intestinal mucosa, and chronic persistent inflammation (9). Moreover, previous studies have suggested that epigenetic alterations and intestinal microecological imbalances are highly relevant risk factors in sarcopenic patients. For one thing, Qiu et al. (6) revealed that the binding of the NF-κB p65 subunit to the myostatin promoter, which could result from hyperammonemia, facilitated the transcription of the myostatin gene. Borja-Gonzalez et al. (10) proposed that increased levels of miR-21, which mediates fibrotic activation of HSCs, could lead to sarcopenia as a result of the effects of H2O2 and pro-inflammatory cytokines. For another, dysbiosis in patients with cirrhosis contributes to the development of sarcopenia. Ponziani et al. (11) suggested that in comparison to age-related sarcopenia, a more predominant mechanism of sarcopenia in cirrhosis is a decrease in the alpha diversity of the intestinal flora. However, the interrelationship between sarcopenia and liver disease in cirrhotic patients, and the role played by intestinal microecological imbalance and epigenetic alterations, have not yet been systematically summarized.

Here, we summarize the mechanism of sarcopenia in cirrhotic patients, with a particular focus on both epigenetics and gut microbiome perspectives, and propose a possible gut microbiota-epigenetics vicious cycle. Moreover, microecological intervention, which is expected to be an effective means to treat or prevent sarcopenia, could improve the prognosis of cirrhotic patients.

## 2. Epigenetic changes are an important genetic basis for the development of liver cirrhosis and sarcopenia

Epigenetics refers to heritable changes in gene expression levels due to non-genetic sequence alterations. Cirrhosis is the outcome of chronic liver disease (CLD) progressing to end-stage. Activation and maintenance of hepatic myofibroblasts (MFs), predominately differentiated from hepatic stellate cells (HSCs), are critical in the pathogenesis of cirrhosis (12). Tsuchida and Friedman (7) indicated that activation and silencing of HSCs are closely associated with epigenetic alterations, including DNA methylation, histone modifications, and miRNA levels, which are also the three main epigenetic alterations associated with sarcopenia.

### 2.1. DNA methylation

The most common type of eukaryotic chromatin DNA methylation is the direct binding of a covalent bond to a methyl group at the fifth position of the cytosine ring in DNA. This variation is mainly present in sequences enriched in cytosine phosphoguanine (CpG) dinucleotides (13). DNA methylation in the promoter region has been shown to cause gene silencing, while the function of methylation in gene body still needs further study (14). For instance, both CAND1 intergenic hypermethylation and vitamin D receptor (VDR) promoter region hypermethylation can repress transcriptional translation of the corresponding genes (15, 16), causing muscle mass reduction by repressing the Akt/mTOR pathway.

### 2.2. Histone modifications

The N-terminal tail of core histones is a common target for histone modifications, which include methylation, acetylation, phosphorylation, and ubiquitination, etc. Depending on the modification mode and site, histone modifications can activate or repress transcription of target genes, thereby affecting gene expression (13). For example, binding of HDAC11 to Myo D can reduce Myo D-dependent histone acetylation near the promoter of the myostatin gene, thus repressing its transcription and causing sarcopenia (17).

### 2.3. microRNAs

miRNAs are short-single-stranded, non-coding RNAs of 19–22 nucleotides, which are initially transcribed in the nucleus and are called pri-miRNAs. Undergoing a series of processes, pri-miRNAs are translocated to the cytoplasm, where they are further processed into miRNA double-stranded bodies, known as mature miRNAs. Mature miRNAs regulate target gene expression by integrating one of the double-stranded bodies into the RNA-induced silencing complex (RISC). Brown and Goljanek-Whysall (18) proposed that miRNAs modulate muscle cell biology by versatile pathways, such as regulating myoblast differentiation and muscle atrophy process, which plays an important role in muscle homeostasis. Changes in the expression levels of miRNAs can cause, exacerbate, or compensate for sarcopenia, for instance, overexpression of miR-21, which contributes to the activation of hepatic stellate cells (HSCs), exacerbates muscle atrophy in denervated muscles.

### 2.4. Other factors

In addition to the factors mentioned above, aging can also lead to a variety of other epigenetic alterations, including histone and heterochromatin loss, histone variants, and ATP-dependent chromatin remodeling, which are probably associated with sarcopenia, especially age-related sarcopenia. However, the role and mechanisms of these epigenetic alterations in cirrhosis-induced sarcopenia need to be further investigated. DNA methylation, histone modifications and miRNA-mediated alterations in cytokine levels are still considered the main epigenetic causes of cirrhotic sarcopenia.

## 3. Dysbiosis is an important microbiological basis for the development of sarcopenia

The total number of microorganisms in the adult gut can reach 10–100 trillion, most of which are members of the *Firmicutes* and *Bacteroidetes* phyla (19). The gut microbiota is now considered one of the key factors regulating host health, and its alteration or dysregulation is strongly associated with a variety of intestinal and extraintestinal diseases (20).

### 3.1. Association between gut microbiota, and intestinal and extraintestinal diseases

Important gastrointestinal diseases such as IBD (21), irritable bowel syndrome (IBS) (22), and colorectal cancers(CRCs) (23) are strongly associated with intestinal microecological disorders. Alterations in gut microbial composition can damage the intestinal barrier, induce tissue inflammation, disrupt protective immunity, and impair intestinal health through mechanisms such as affecting cellular immunity and the enteric nervous system (ENS), and decreasing levels of short-chain fatty acids (SCFA) and indole metabolites.

The gut microbiota is also extensively associated with extraintestinal tissue and organ function. Various studies have demonstrated that diseases such as chronic liver disease, pancreatic adenocarcinoma, and diabetes are closely related to dysbiosis (20). Albillos et al. (24) proposed the gut-liver axis, a bidirectional relationship between gut and its microbiota and the liver. The portal system is the channel of connection between gut and liver, and intestinal mucosa and vascular barrier are the functional and anatomical basis for the interaction between gut and liver. The intestinal microbiota plays an important role in maintaining the homeostasis of the gut-liver axis, and its composition is also influenced by the liver. Disturbed intestinal microecology can be an external manifestation of the dysregulation of the gut-liver axis after cirrhosis.

### 3.2. Gut microbiota correlates with sarcopenia

Gut microbes can be directly or indirectly correlated with musculoskeletal dysfunction. The existence of the gut-muscle axis and gut-bone axis was demonstrated in previous studies (11, 25, 26). Gut microbes can be directly or indirectly correlated with musculoskeletal dysfunction. The existence of the gut-muscle axis and gut-bone axis was demonstrated in previous studies (11, 25, 26). To be more specific, the gut-liver-muscle axis is a complex network which involves the persistent chronic inflammation, reduction of protective metabolites, and the consequent systemic alteration of pro-inflammatory mediators.

#### 3.2.1. Chronic inflammation

Gut microecological dysbiosis can lead to systemic low-grade inflammation through diverse pathways. Gut flora has been found to be involved in the metabolism of several substances, such as TMAO, which can activate mitogen-activated protein kinase (MAPK) and NF-κB signaling pathways in endothelial and smooth muscle cells (27). Besides, aging and many diseases such as liver cirrhosis can lead to intestinal mucosal barrier dysfunction, allowing intestinal opportunistic pathogenic bacteria or bacterial products such as lipopolysaccharide (LPS) to enter the blood circulation. All the pathways and substances mentioned above could activate the inflammatory response, leading to monocyte recruitment or an increase in pro-inflammatory cytokines such as IL-6, IL-8, and TNF-α, causing persistent chronic inflammation, which is not only a vital pathogenic factor in causing muscle loss, but also an important molecular link between dysregulated intestinal microecology and sarcopenia (28).

#### 3.2.2. Intestinal flora metabolite

Dysregulated intestinal microbiota reduces SCFA production, short-chain fatty acids produced by fermentation of indigestible foods by intestinal microbes, which are powerful anti-inflammatory molecules that modulate host immunity and are good for maintaining intestinal health (29). SCFA have been proved to be an essential linking factor between the gut microbiota and skeletal muscle, preventing lipid deposition in skeletal muscle by affecting substance metabolism, and also sensitizing skeletal muscle cells to insulin, which regulates skeletal muscle function (30).

#### 3.2.3. Pathogen-associated molecular patterns

PAMPs include LPS, flagellin, peptidoglycan, etc. It has been established that altered intestinal microbiota led to an increased chance of intestinal infection in elderly patients (26). After infection, PAMPs bind to the Toll-like receptor 2 (TLR2), TLR4, and TLR5 on the gut mucosal surface, activating the TLRs/NF-κB pathway (31). NF-ĸB factors can bind to the muscle growth inhibitor promoter and mediate the transcription of muscle growth inhibitor (6), negatively regulating muscle growth and causing impaired proliferation and differentiation of myogenic cells.

### 3.3. Dysbiosis caused by cirrhosis accelerate the appearance of sarcopenia

Cirrhosis can contribute to impaired intestinal barrier function, microecological disorders, and chronic low-grade inflammatory diseases, all of which are thought to be important factors in cirrhosis leading to sarcopenia. Ponziani et al. (11) conducted a study to explore the predominant factors involved. They found that, as opposed to sarcopenia due to aging, there was no difference in the degree of altered intestinal barrier integrity or local inflammation between patients in cirrhosis with or without sarcopenia. This indicated that intestinal microecological disorders may be a more relevant factor in cirrhosis-inducing sarcopenia than intestinal barrier damage.

Consequently, they further investigated the alterations in the gut microbiota due to cirrhosis and found that the alpha diversity of gut microbiota was reduced in cirrhotic patients with sarcopenia compared to both cirrhotic patients without sarcopenia and patients with sarcopenia alone (control group). Furthermore, compared with the control group, the variation of alpha diversity of gut microbiota in sarcopenic cirrhotic patients mainly showed the characteristics of cirrhotic patients, and the increased abundance of *Klebsiella* and *Streptococcus*. In contrast, compared to cirrhotic patients without sarcopenia, the abundance of *Methanobrevibacter*, *Pseudomonas*, *Proteus*, and *Akkermansia*, which were beneficial to intestinal metabolism, was lower in cirrhotic patients with sarcopenia. Moreover, *Methanobrevibacter* and *Proteus* are important bacteria that promote SCFA production, whose decrease leads to SCFA deficiency. As mentioned previously, reduced SCFA production is an important factor affecting skeletal muscle function, and its deficiency can accelerate skeletal muscle atrophy and dysfunction. Simultaneously, the reduction of anti-inflammatory microorganisms such as *Akkermannia* (32) in the gut causes overproduction of several cytokines in patients with cirrhosis, most of which have been demonstrated to be correlated with sarcopenia, such as IL-6 and CRP.

## 4. Synergistic or accelerating roles of cirrhosis in the mechanism of sarcopenia

### 4.1. Overview of the pathophysiology of liver cirrhosis

Patients with cirrhosis have pathophysiological changes including reduced insulin-like growth factor 1 (IGF-1) level (33), oxidative stress (34, 35), and upregulation of inflammatory factors such as IL-6 (36).

Compared with normal subjects, serum IGF-1 level is much lower and IL-6 levels were much higher in patients with cirrhosis, and there was a negative correlation between levels of IGF-1 and IL-6. All these alterations are closely related to the patients’ liver dysfunction. For instance, elevated IL-6 levels are associated with decreased hepatic clearance of cytokines. Furthermore, inflammatory factors like IL-6 activate the NF-κB and ubiquitin-proteasome systems and reduce IGF-1 levels in plasma and muscle. In addition, levels of IGF-1 are also affected by growth hormone (GH). There is a GH-liver-IGF-1 axis existing in normal human body, in which liver is the main target organ of GH, and GH can regulate IGF-1 production in liver. However, the liver’s ability to synthesize GH receptors is declined and GH resistance is observed when liver function is impaired in cirrhosis, resulting in increased serum GH levels and decreased IGF-1 levels (33). Another common pathophysiologic change in cirrhosis is oxidative stress, which has been confirmed to play a significant role in fibrogenesis and toxic liver damage in cirrhosis (34). In addition, many pathogens involved in fibrogenesis induce free radical reactions (35), causing oxidative stress.

### 4.2. Pathophysiological changes in cirrhosis influence the process of epigenetic alterations leading sarcopenia

#### 4.2.1. CAND1 hypermethylation and IGF-1 affect AKT/mTOR pathway

CAND-1 intergenic hypermethylation indirectly enhances SCF complex action by blocking the protein-increasing effect of IGF1/insulin receptor substrate 1 (IRS1)/PI3K/Akt/mTOR pathway through IRS1 ubiquitination (15, 37, 38).

CAND1 is a 120 kDa heat repeat protein that inhibits the assembly of the SCF E3 complex (37), which functions primarily in the IGF1/IRS1/Akt/mTOR pathway. Fbxo40, a component protein of the SCF E3 complex, binds to the Skp1-Cul-1-Rbx1 complex, targeting IRS1 for ubiquitinated degradation, thereby blocking IGF1/IRS1/Akt/mTOR pathway-mediated protein increase (38).

Intergenic hypermethylation of the gene encoding CAND-1 has been revealed to down-regulate its transcription and translation (15), which diminishes the inhibitory effect of CAND-1 on the SCF E3 complex and indirectly enhances the action of the SCF E3 complex, leading to increased protein degradation and atrophy and malfunction in skeletal muscle.

Extensive studies have demonstrated that serum IGF-1 levels are reduced in cirrhotic patients. Saeki et al. (39) have demonstrated that low IGF-1 levels remained an independent risk factor for sarcopenia in patients with cirrhosis after controlling for other factors significantly associated with sarcopenia, such as age and BMI, and that the lower the IGF-1 levels in cirrhotic patients, the higher the prevalence of sarcopenia and muscle loss. In the IGF1/IRS1/Akt/mTOR pathway, decreased IGF-1 induced by liver disease and decreased ubiquitination of IRS-1 induced by epigenetic alterations block the pathway upstream and downstream respectively, leading to the development of sarcopenia.

#### 4.2.2. Histone deacetylation and cirrhosis affect myostatin production

HDAC11 inhibits myogenic cell differentiation by targeting histones adjacent to the myostatin gene promoter through binding to Myo D (40).

Differentiation of myogenic cells into myotubes is important for the development of muscle-specific structures and contractile function. It is primarily regulated by two families of transcription factors, myogenic regulatory factor (MRF) and cardiomyocyte enhancer factor 2 (MEF2). Myo D, a member of MRF, binds to the promoters of MEF2C and myostatin to promote muscle-specific gene expression and myotube formation (40). HDAC11, known as histone deacetylase 11, is the only member of the fourth subfamily of HDACs and is highly expressed in skeletal muscle (17). Byun et al. (40) found that HDAC11 deacetylates histones H3 and H4 and binds to Myo D and MEF2, targeting the promoters of muscle-specific genes as well as Myo D and MEF2. This can reduce Myo D-dependent acetylation of histones near the promoters of MEF2C and myostatin genes, which in turn inhibits myostatin transcription and myogenic cell differentiation, thus causing sarcopenia.

Yang et al. (41) showed significant upregulation of gene expression, such as HDAC11, in patients with hepatocellular carcinoma (HCC), especially those who have a background of cirrhosis, contributing to the diagnosis of HCC. And the upregulation of HDAC11 expression facilitates its binding to Myo D, which cooperates with epigenetic alterations to promote the development of sarcopenia.

#### 4.2.3. miR-532-3p and inflammation affect the TLR4/NF-κB1 signaling axis

Suppression of miR-532-3p expression mediated by TLR4 and NF-κB1 during inflammation allows BAK1 accumulation and leads to muscle cell apoptosis (42).

miR-532-3p, a miRNA targeting the 3′ end of the BAK1 gene, significantly represses the expression of BAK1, the accumulation of which can lead to the release of cytochrome C from mitochondria and activation of the endogenous apoptotic pathway (43). Chen et al. (42) found that miR-532-3p was the most significantly down-regulated miRNA in patients with sarcopenia and proposed that its down-regulation was mainly due to activation of TLR4/NF-κB1 signaling axis in response to inflammatory stimuli. The activation of the axis allows NF-κB1 to translocate from the cytoplasm to the nucleus and bind to miR-532-3p promoter. In turn, downregulation of miR-532-3p attenuates its inhibitory effect on BAK1, prompting muscle cell apoptosis and causing sarcopenia.

Elevated pro-inflammatory factors such as IL-6 and TNF-α are known to be important causes of muscle atrophy, protein catabolism, and inhibition of muscle synthesis (44). In addition, patients with cirrhosis often have systemic inflammation and significantly high levels of pro-inflammatory factors such as IL-1β, IL-6, and TNF *in vivo* (45). Moreover, TLR4 is a key factor in the progression to acute or chronic liver failure in patients with cirrhosis, and inhibition of TLR4 signaling pathways can not only reduce the degree of organ damage but improve patient prognosis (46). These suggest that cirrhosis can cause a decrease in miR-532-3p by causing inflammation *in vivo* and activating TLR4, leading to the development of sarcopenia.

#### 4.2.4. VDR hypermethylation and cirrhosis affect AKT/mTOR pathway

Hypermethylation of the VDR promoter region decreases VDR expression, which reduces AKT/mTOR anabolic signaling and leads to low muscle mass and strength (16, 47).

Vitamin D is a fat-soluble steroid derivative, which mainly functions in the human body as vitamin D2 and vitamin D3. Vitamin D3 is hydroxylated *in vivo* in two steps to the biologically active 1, 25 (OH) _2_D_3_, which binds to its nuclear receptor VDR and induces elevated blood calcium and phosphorus, leading to bone mineralization and remodeling (48).

Numerous papers have been published linking vitamin D and vitamin D receptors to aging-related diseases such as osteoporosis and low muscle mass. Overexpression of the VDR gene in rats, which encodes the vitamin D receptor in skeletal muscle, leads to skeletal muscle hypertrophy by mechanisms that increased AKT/mTOR anabolic signaling, muscle protein synthesis, ribosome biogenesis, and satellite cell activation (47). Meanwhile, the hypermethylation of the VDR promoter region in the sarcopenic group has been confirmed by comparing differences in DNA methylation associated with sarcopenia in blood samples from control and elderly women with sarcopenia (16). Consequently, muscle atrophy in these elderly women may be attributed to decreased expression of VDR, which results in low muscle mass and strength.

Skeletal muscle of mice with cirrhosis induced by bile duct ligation (BDL) had myasthenia and a significant decrease in the expression levels of Akt and mTOR, while the expression of myostatin, which negatively regulated the activity of the Akt–mTOR axis, was increased (49). This further demonstrates that the reduction in protein synthesis associated with the Akt–mTOR axis is one of the main mechanisms leading to muscle loss in BDL mice.

#### 4.2.5. Oxidative stress and histone hypomethylation

Oxidative stress reduces histone H3K9 methylation levels and promotes muscle cell apoptosis (50–53).

Mecocci et al. (50) found that aging could cause increased oxidative damage to skeletal muscle *in vivo*, and that *in vitro* treatment of cultured skeletal muscle cells with H2O2 promoted the expression of apoptosis-related genes and induced apoptosis (51).The possible mechanism for this is that the reactive oxygen species generated by H2O2 can promote the binding of histone H3K9 demethylase (KDM3A) to the E2F1 promoter. Moreover, KDM3A can remove dimethyl H3K9 from the target gene promoter, which activates the target gene transcription (52). Therefore, oxidative stress caused by reactive oxygen species decreases H3K9 methylation level on E2F1 gene promoter and upregulates E2F1 expression level. In contrast, reducing the level of KDM3A using interfering RNA significantly attenuated the reactive oxygen species-induced upregulation of E2F1 expression. It is suggested that KDM3A may be involved in reactive oxygen species-induced transcriptional activation of E2F1 by modifying histones (53), which further leads to apoptosis through multiple mechanisms. For instance, through the p53-dependent pathway, E2F1 activates p14/p19 to inhibit p53 degradation and p53 overexpression inhibits downstream CDK activity, thereby interrupting cell cycle progression; while through the p53-independent pathway, E2F1 induces apoptosis by enhancing the activity of regulatory genes p73, CASP-3, and CASP-7, etc.

Oxidative stress in cirrhotic patients can increase reactive oxygen species *in vivo*, resulting in increased progression of this pathway.

#### 4.2.6. BET protein and upregulated IL-6 affect the IL-6/AMPK/FoxO3 axis

The bromodomain terminal (BET) family of proteins, including BRD2, BRD3, BRD4 and BRDT, are epigenetic readers of gene transcriptional regulation (54). Bromo dopamine-containing protein 4 (BRD4) is the most studied member of the BET family, which interprets the epigenetic code by binding to acetylated histones and non-histones, regulating a variety of cellular processes including cell cycle, cell differentiation and cell proliferation (55). BETs were found to coordinate the expression of genes associated with muscle atrophy through direct occupancy of catabolic genes by BRD2 and activation of the IL-6/AMPK/FoxO3 axis, leading to the development of sarcopenia (56, 57). BRD4 is directly connected to the regulatory regions of key catabolic genes in skeletal muscle. In addition, BRD4 recruitment was significantly increased during muscle atrophy. Blocking BET reduces systemic IL-6 levels and prevents AMPK activation in muscle, while AMPK in its active form contributes to FoxO3 (a kind of transcription factor) phosphorylation, facilitating transcriptional activation of catabolic genes.

Upregulation of inflammatory factors such as IL-6 in cirrhosis enhances the activation of the IL-6/AMPK/FoxO3 axis.

The changes and pathways discussed can be seen in Table 1.

**Table 1:** Epigenetic changes, functions, target genes, and pathways associated with cirrhotic sarcopenia.

Epigenetic changes	Functions	Target genes	Key factors / pathways
CAND1 intergenic hypermethylation	Direct down-regulation of CAND1 repression	CAND1 (15)	–
Deacetylation of histone in myostatin promoter region	Direct down-regulation of myostatin	Myostatin, MEF2C (40)	HDAC11 Myo D
Decreased miR-532-3p levels	Indirect down-regulation of BAK1 repression	BAK1 (42)	TLR4/NF-κB1 signaling axis
Hypermethylation of the VDR	Direct down-regulation of VDR repression	VDR (16)	–
Histone H3K9 hypomethylation in the E2F1 gene	Direct up-regulation of E2F1 repression	E2F1 (52)	KDM3A
BET binding to histones of catabolic genes	Indirect up-regulation of atrogene expression	Catabolic genes (57)	IL-6

## 5. Epigenetic modifications and dysbiosis constitute a vicious cycle, accelerating sarcopenia in cirrhotic patients

### 5.1. Intestinal microecological disorders in cirrhotic patients play a facilitating role in epigenetic pathways

Gut microecological disorders lead to a decline in probiotics such as *Lactobacillus* and beneficial microbial products such as SCFA, causing inflammatory responses (58) and oxidative stress in the body, which in turn affects epigenetic pathways.

SCFA mainly include acetate, propionate, and butyrate, which are produced by the fermentation of colonic microflora. It has been revealed that NaB, a kind of butyrate, can depress the effects of HDAC (59) in muscle tissue and increase the acetylation level of histones near the myostatin gene promoter. This increases the transcriptional activity of the myostatin gene, which improves muscle mass and cross-sectional area in aged mice (60). Conversely, NaB deficiency accelerates the epigenetic pathway mediated by HDAC11 described previously, causing sarcopenia.

*Lactobacillus,* which has been shown to directly elevate IGF-1 levels (61), may have a critical role in regulating the GH/IGF-1 axis. Meanwhile, *Lactobacillus* is one of the producers of SCFA, which increases IGF-1 levels in serum, adipose tissue, and liver of mice (62). Consequently, the effects of normal intestinal flora, such as *Lactobacillus*, are diminished in pathological conditions, directly and indirectly leading to a reduction in IGF-1 levels and promoting the above-mentioned CAND-1 intergenic hypermethylation-mediated epigenetic pathway. Likewise, dysbiosis can affect other epigenetic pathways listed previously by promoting inflammatory responses and oxidative stress.

### 5.2. Abnormal epigenetic modifications in cirrhotic patients may contribute to dysbiosis

It has been confirmed that gene modifications can affect intestinal microbes, for example, Alengha et al. (63) found significant changes in intestinal microbiota composition in mice that lacked HDAC3 in intestinal epithelial cells (IECS), including significant increases in *Aspergillus* and *Mycobacterium*. Cortese et al. (64) found that *in utero* epigenomic changes constrain the colonization of early microbiota. However, the current literature is mostly limited to the study of abnormal gene modifications in intestinal cells. Whether genetic modifications in myoblasts during the pathogenesis of sarcopenia can affect the microbiome needs further investigation.

**Figure 1:**
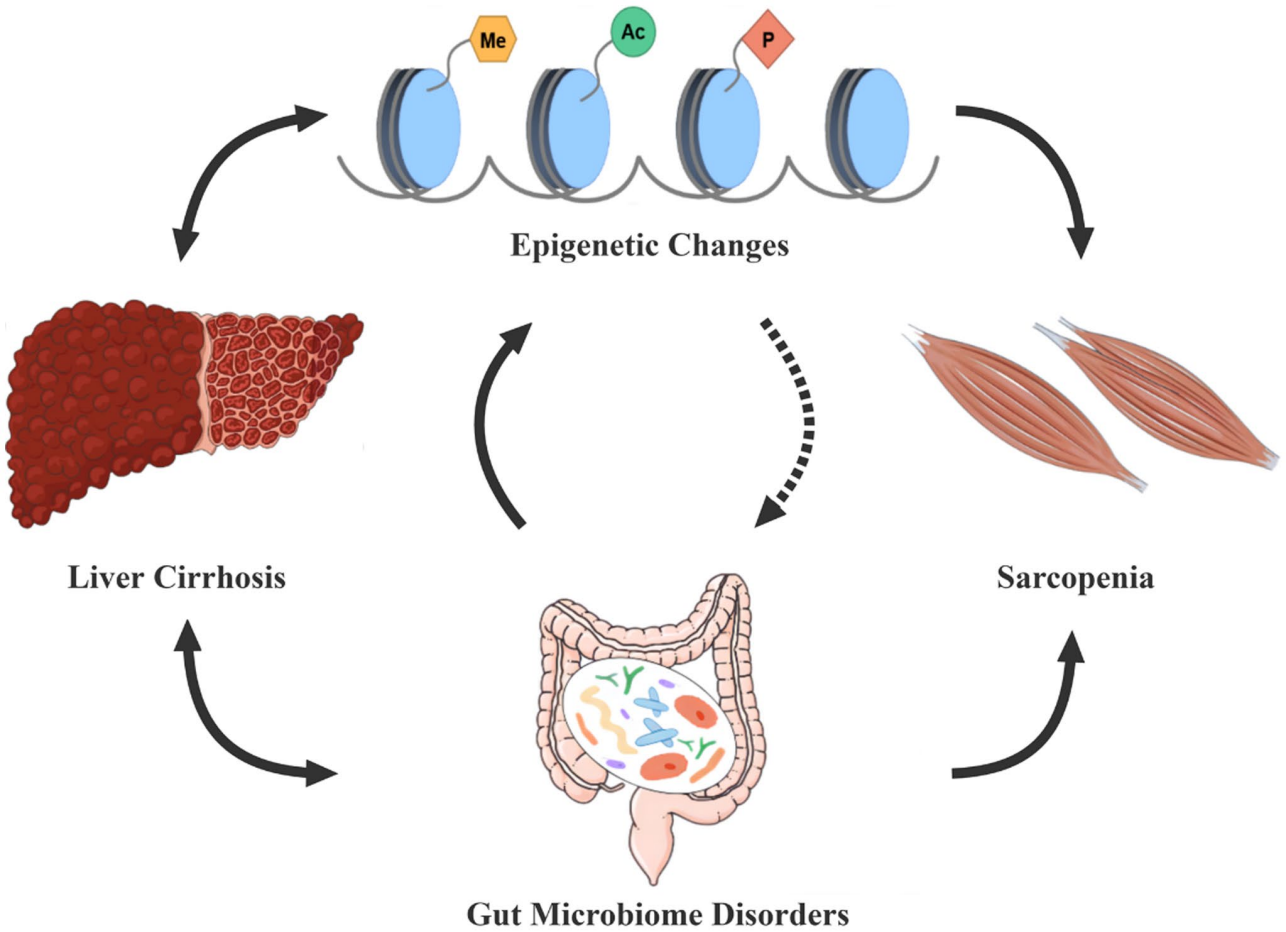
Epigenetic modifications-dysbiosis vicious circle in cirrhosis patients with sarcopenia. Gut microecological disorders and epigenetic changes are often present in cirrhotic patients, both of which seem to interact in a vicious circle, thus constituting an important cause of the development of sarcopenia. The dashed line in the figure indicates that further studies are needed to demonstrate the effect of epigenetic modifications on gut microbes. Conversely, dysbiosis and epigenetic alterations can also enhance the progression of cirrhosis.

Therefore, there may be a vicious cycle between epigenetic modifications and dysbiosis, accelerating sarcopenia in cirrhotic patients (Figure 1).

## 6. Microbiome-based therapies promise to break the vicious circle in sarcopenia

### 6.1. Overview of microbiome-based therapies

**Figure 2:**
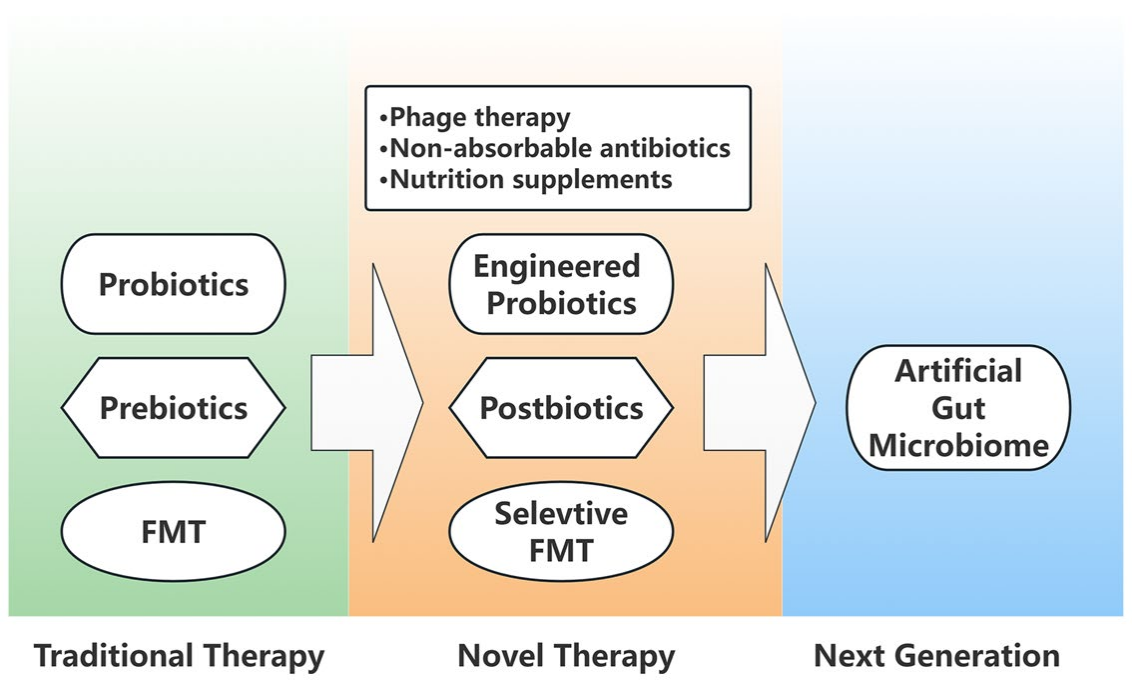
Generation diagram of intestinal microecological interventions. Traditional microbial therapies include probiotics, prebiotics, and FMT (Fecal microbiota transplantation), which are optimized in novel interventional therapies as engineered probiotics, postbiotics, and selective FMT. In addition, novel therapies also include phage therapy, non-absorbable antibiotics, and nutritional supplements. The next generation of artificial gut microbiome is also attracting a wide interest.

Since its introduction into clinical practice, intestinal microecological interventions have developed at a rapid pace and can be summarized in the following three main stages of renewal (Figure 2).

#### 6.1.1. Traditional therapies

Traditional therapies include fecal microbiota transplantation (FMT), probiotics and prebiotics. The aim of FMT is to replace the entire gut microbiota with the stool from a healthy donor (65). Since all microorganisms are transplanted together, the effectiveness and safety remain to be further validated. Moreover, FMT is gradually being replaced by selective microbiota transplantation. Colonizing probiotics, including yeast, *Bacillus* probiotic strains, *Clostridium butyricum*, *Lactobacillus*, *Bifidobacterium*, and *Actinobacillus*, etc., have beneficial effects on the modulation of flora composition. Prebiotics cannot be digested or absorbed by the host but selectively promote metabolism and proliferation of beneficial bacteria in the body.

#### 6.1.2. Novel means of intervention/precision microbiome-centered therapies

Postbiotics: Postbiotics are defined as “inactivated bacteria and/or bacterial components helpful to the host” (66). With well-defined chemical structures, postbiotics act as a powerful immune activator that destroys existing harmful bacteria and helps beneficial bacteria to proliferate;

Engineering bacteria: Engineered bacteria are genetically engineered probiotics that can be selected for their potential benefits through large-scale genomic screening and advanced sequencing technologies (67). For example, specific health-promoting genes associated with improved gut colonization, host-bacterial interactions, immunomodulation, antimicrobial activity or pathogen suppression can be selected (68), further enhancing the beneficial effects of probiotics. In medical applications, engineered probiotics constructed by synthetic biology techniques can improve the intestinal microenvironment by expressing specific products such as enzymes, cytokines, antimicrobial peptides, hormones, and antibodies. As a result, engineered probiotics are more controllable, safe, and specific than traditional interventions;

Other means: interventions such as bacteriophage (targeted killing of known pathogenic bacteria) (65), non-absorbable antibiotics (69), nutritional supplements, and selective flora transplantation Can function In regulation.

#### 6.1.3. Next-generation microbiome-based therapies/synthetic intestinal microbiota

The idea of synthetic intestinal microbiota emerged due to unstable effects of traditional interventions and the weak resistance of single engineered probiotics to disruption. Synthetic microbiota has been extensively applied in basic research in the fields of energy development and environmental protection. However, research on synthetic microbiota targeting intestinal microecology is still in its infancy. Sixteen key human intestinal bacteria have been used to build a synthetic microbiota MDb-MM (mucin and diet based minimal microbiome) to study the ecological and metabolic interactions of human intestinal microbiota (70). Two microbiota combinations, GUT-103 (containing 17 strains) and GUT-108 (containing 11 strains), were designed to target intestinal dysbiosis in patients with IBD, which reduced pathogenic bacteria, expanded the population of beneficial bacteria, produced metabolites for mucosal healing and immune modulation, and reduced inflammatory factors (71). However, the safety and efficacy of the technology need to be further explored.

### 6.2. Corrective effect of microbiome-based interventions on epigenetic abnormalities

#### 6.2.1. Regulation on IGF-1

The presence of gut microbiota is positively correlated with growth hormone and IGF-1 levels, thereby affecting overall growth and development of the host (61). Lahiri et al. (72) showed that serum IGF-1 levels in germ-free mice were lower than in pathogen-free mice with gut microbiota, and that their skeletal muscle atrophied and transcription of genes related to skeletal muscle growth and mitochondrial function reduced. In contrast, increased skeletal muscle mass and enhanced muscle oxidative metabolism were observed in germ-free mice when gut microbiota from pathogen-free mice were transplanted into germ-free mice. Similarly, counts of *Lactobacillus* and *Faecalibacterium* increased and growth parameters (including total body weight, plasma GH, and IGF-1 levels) were elevated in suckling pigs after FMT (73). Further, supplementation with a single probiotic such as *Lactobacillus* can also increase IGF-1 levels (as described above).

#### 6.2.2. Regulation on vitamin D

Microbiota can affect vitamin D metabolism. Some bacteria express enzymes involved in hydroxylation of steroids and are therefore able to process and activate vitamin D (74). Bacteria CYP105A1 from *Streptomycesgriseolus* converts vitamin D3 into 1,25(OH)2D3 in two independent reactions, suggesting that bacterial function is equivalent to that of vitamin D metabolizing enzymes (75). Therefore, rational regulation of the gut microbiota may promote vitamin D metabolism, increase VDR expression (76), and resist the effects of VDR gene hypermethylation.

#### 6.2.3. Regulation on oxidative stress

Prebiotics may regulate oxidative stress in cirrhosis to some extent. Prebiotic-treated mice exhibited more intact intestinal tight junctions, improved intestinal permeability, reduced plasma cytokine and LPS levels, and reduced hepatic oxidative stress expression and inflammatory markers (77). This effect may be achieved by IL-10 secretion and TLR4 expression to restore the phagocytic ability of neutrophils. Prebiotics can also reduce intestinal wall permeability, bacterial translocation and endotoxemia, and limit oxidative stress and inflammatory injury of the liver.

#### 6.2.4. Regulation on inflammatory reactions

Many studies have confirmed that probiotics and probiotics have certain anti-inflammatory effects. For example, pro-inflammatory cytokines such as IL-6 in mice were reduced by treatment with colitis with camel milk rich in probiotics and prebiotics (78). Probiotics can significantly reduce the contents of inflammatory factors and lipopolysaccharide in serum (79). A probiotic formulation of the predominantly SCFA-producing bacterium, *Pseudomonas putida*, improves liver metabolism and reduces systemic inflammatory responses in mice. Interestingly, Forslund et al. (80) found that the combination of diuretics and antihypertensive drugs (beta blockers) was associated with an increase in the number of health-promoting bacteria *Roseburia*, which could break down dietary fiber in plant foods and convert it into butyric acid, and might play a role in reducing inflammation and regulating epigenomes in cirrhosis. Altogether, in patients with cirrhosis complicated by sarcopenia, microbiome-based interventions counteract the activation of the IL-6/AMPK/FoxO3 axis due to IL-6 upregulation on the one hand, and reduce the disruption of intestinal barrier function and intestinal bacterial translocation caused by cirrhosis on the other hand, thereby reducing bacterial endotoxin levels and alleviating systemic inflammatory response.

**Figure 3:**
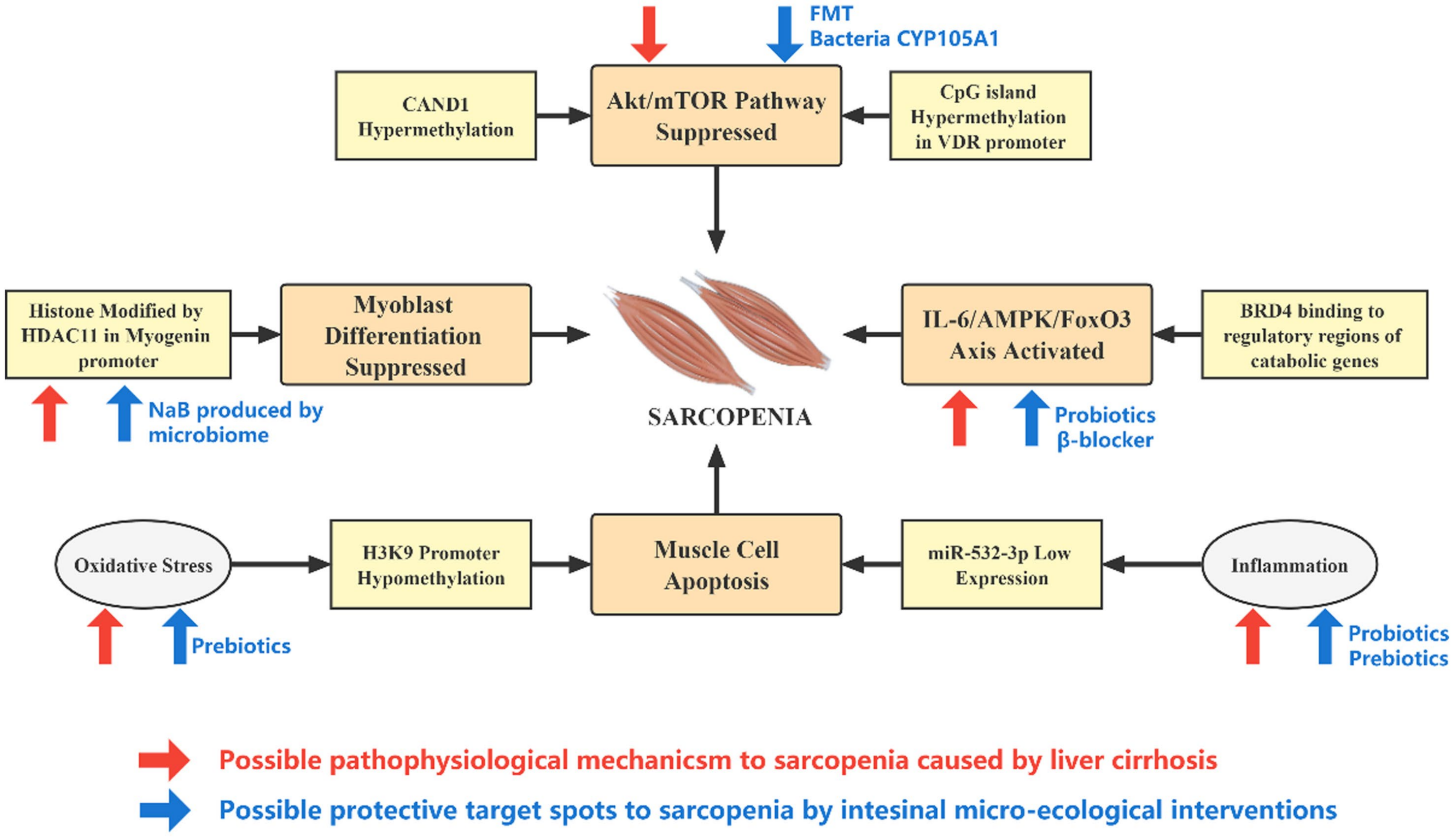
Effect of epigenetic alterations and microecological interventions on the development of sarcopenia in patients with cirrhosis. The pathophysiological changes caused by liver cirrhosis and intestinal micro-ecology can mediate or slow down the occurrence of sarcopenia through epigenetic pathways at different targets. Cirrhosis is associated with inflammation, oxidative stress, elevated IL-6, and IGF-1, while intestinal micro-ecological interventions may directly counteract these effects. In addition, the expression of genes such as HDAC11 is significantly upregulated in cirrhosis, and microbial product NaB may hinder its action. The activity of the Akt–mTOR axis is reduced in cirrhosis, and a rational micro-ecological intervention may promote vitamin D metabolism, increase VDR expression and enhance the activity of Akt–mTOR axis from upstream.

To sum up, microbiome-based therapies may combat sarcopenia through multiple potential pathophysiological mechanisms, breaking the vicious cycle of epigenetic- dysbiosis and improving intestinal microecology (Figure 3).

## 7. Discussion

Sarcopenia, often recognized as an age-related progressive skeletal muscle disorder, has received burgeoning attention. In recent years, decreased muscle mass and loss of muscle function have also been found to occur in various chronic disease processes, with liver cirrhosis being one of the typical examples. Researchers have revealed that comorbid sarcopenia can impact the prognosis of patients with cirrhosis, while the mechanism of the link between cirrhosis and sarcopenia has been the subject of much discussion. In addition to nutrition and physical activity level, as is commonly considered the etiology of cirrhosis related sarcopenia, we believe that there is a deeper pathophysiological connection between liver cirrhosis and sarcopenia. Here we innovatively combine genetic modification and intestinal microecology in the background of liver cirrhosis, focusing on the potential pathways by which pathophysiological alterations in liver cirrhosis promote the development of sarcopenia. Furthermore, there may also be an interaction between epigenetic modifications and dysbiosis, and the two constitute a vicious circle leading to sarcopenia.

In patients with liver cirrhosis, early intervention and prevention of sarcopenia is of great importance, which can contribute to the improvement in prognosis. Reasonable and effective microbiome-based therapies could, on the one hand, prevent inflammatory reactions and SCFA reduction caused by intestinal microecological dysregulation from affecting skeletal muscle quality. On the other hand, they may play a role in targeting and regulating the “brakes” at different stages of epigenetic pathways, thus delaying the development of sarcopenia. Microbiome-based therapies continue to evolve from traditional non-targeted interventions to precision therapies and to next-generation microbiome-based therapies with synthetic flora, showing wide promise for future interventions in sarcopenia. Overall, microecological interventions may be one of the effective preventive and therapeutic strategies, while more large-scale clinical trials are still needed to support the specific treatment regimens and efficacy.

## Author contributions

J-qX: Writing – original draft, Writing – review & editing. Y-kP: Writing – original draft, Writing – review & editing. J-xZ: Conceptualization, Writing – review & editing. S-xD: Conceptualization, Supervision, Writing – review & editing. L-sX: Funding acquisition, Writing – review & editing.
